# Livestock owners’ anthrax prevention practices and its associated factors in Sekota Zuria district, Northeast Ethiopia

**DOI:** 10.1186/s12917-020-2267-0

**Published:** 2020-02-03

**Authors:** Kibeb Seid, Atsede Mazengia Shiferaw, Nurhusien Nuru Yesuf, Terefe Derso, Mekonnen Sisay

**Affiliations:** 1 Sekota Town agricultural office, Sekota Zuria district, Wag-Himra Zone, northeast, Amhara Region State, Ethiopia; 2 Department of Health Informatics, Institute of Public Health, College of Medicine and Health Sciences, University of Gondar, Gondar, Ethiopia; 3 Department of Surgical Nursing, School of Nursing, College of Medicine and Health Sciences, University of Gondar, Gondar, Ethiopia; 4 Department of Human Nutrition, Institute of Public Health, College of Medicine and Health Sciences, University of Gondar, Gondar, Ethiopia

**Keywords:** Anthrax prevention, Practices, Livestock owners, Ethiopia

## Abstract

**Background:**

In Ethiopia, the second most prioritized of the zoonotic diseases next to rabies is anthrax. About 50.6% of anthrax cases and 33.3% of deaths of livestock have been reported from Wag-Himra Zone, where appropriate anthrax prevention practices are not implemented by the owners of the animals. Thus, the aim of this study was to determine the extent of appropriate anthrax prevention practices of livestock owners and associated factors in Sekota Zuria district, northwest Ethiopia.

**Results:**

Twenty-five percent (95% CI: 25.2, 26.1%) of the livestock owners implemented appropriate anthrax prevention. Three quarters (74%) of the owners consumed infected meat; more than three quarters (78%) used the skins and hides of animals found dead with anthrax. The odds of appropriate anthrax prevention practices were higher among livestock owners with positive attitude (AOR = 4.16, 95% CI: 2.72, 6.37), who received health education (AOR = 2.00, 95% CI: 1.21, 3.28) and owners who lived in urban areas (AOR = 2.62, 95% CI: 1.43, 4.77) compared to their counterparts. Ability to read and write (AOR = 2.76, 95% CI: 1.74, 4.37), and primary (AOR = 3.6, 95% CI: 1.74, 4.37) or secondary school and above education (AOR = 4.24, 95% CI: 1.61, 11.13) were significantly associated with appropriate anthrax prevention practices.

**Conclusion:**

In Sekota Zuria district, only one quarter of the livestock owners were aware of appropriate anthrax prevention practices. Thus, implementing effective health education and creating positive attitude are vital to improve anthrax prevention practices in the area.

## Background

Anthrax is a zoonotic bacterial disease caused by *Bacillus anthracis* [1].When *B. antharcis* are exposed to air, they rapidly sporulate to form very persistent spores [2]. The spores (i.e., dormant stage) are resistant to heat and chemical disinfectants and may persist and remain viable in soil for several decades [2, 3]. Herbivorous and wild mammals are the most commonly affected groups through the ingestion or inhalation of spores while grazing. Carnivores living in the same environment may be infected by consuming infected animals [4].

The disease is transmitted to humans via direct or indirect contacts with infected animals and their products, like hides or wool as well as by the ingestion and inhalation of spores [5]. Human cases usually develop after exposure to infected animals and their tissues. Farmers, butchers, veterinarians, shepherds and farm workers are at a great risk for exposure to infected materials. The disease, often considered occupational in developing countries [6, 7], is associated with rural areas or agricultural production [8].

Globally, 10,000–100,000 human anthrax incidences occur annually with a significant number of cases from developing countries, like Chad, Ethiopia, Zambia, Zimbabwe and India. At the moment, anthrax stands second only to rabies among the zoonotic diseases which are dealt with by one health approach [9]. The federal ministries of health and agriculture surveillance data of Ethiopia (2009–2013) reported a total of 5197 human and 26,214 animal anthrax cases. The highest human cases of 6.7 and 2.3% per 100,100 population [10] were reported from Tigray and Amhara regions, respectively. About 2602 anthrax cases and 18 deaths were reported from Amhara region between 2010 and 2014; 50.6% of the cases and 33.3% of the deaths were from Wag-Himra Zone [11].

The culture of raw meat consumption combined with the low level of awareness about anthrax have increased the risk for contracting the disease in Ethiopia [12]. Additionally, most of the people of Wag-Himra support their life by cattle, sheep and goat husbandry which potentially exposes them to anthrax due to close contacts with livestock, use of hides and skins for different purposes and the consumption of the meat of animals that died from unknown cases with their families and neighbours [11]. Livestock owners’ knowledge and appropriate practices of prevention play a key role in the control of the disease both in humans and animals. Studies which could provide updated evidences about anthrax prevention practices of livestock owners have not been done in the area and in the region. Hence, the results of this study would provide important information to concerned bodies to help design appropriate and feasible interventions. Therefore, this study aimed at assessing the extent of livestock owners’ anthrax prevention practice and associated factors in Sekota Zuria district, northeast Ethiopia.

## Results

### Socio-demographic and disease experience characteristics

In this study, a total of 800 livestock owners participated with a response rate of 94.8%. About three quarters (78.5%) of the participants were male; over half (53.3%) were illiterate and approximately 55% were in the age group of 18–30 years. A great majority (88.9%) were farmers, 87% of whom lived in rural areas. In previous years about 21% of the family members and 49% of their animals were infected by the disease (Table 1).

**Table 1:** Socio-demographic characteristics and previous disease experience of study participants, Sekota Zuria district, northeast Ethiopia, 2018

Variables	Frequency	Percent (%)
Sex		
Female	172	21.5
Male	628	78.5
Age in years		
18–30	131	16.4
31–41	308	38.5
> = 42	361	45.1
Educational status		
Unable to read and write	428	53.5
No formal education, but able to read and write	217	27.1
Completed primary	109	13.6
Completed secondary or above	46	5.8
Occupational status		
Farmer	711	88.8
Merchant	43	5.4
Government employee	19	2.4
Student	27	3.4
Residence		
Rural	701	87.6
Urban	99	12.4
Health education on anthrax prevention		
Yes	125	15.6
No	675	84.4
Regulatory control/anthrax surveillance		
Yes	240	30
No	560	70
Previous anthrax infection experience in humans		
Yes	168	21
No	632	79
Previous anthrax infection experience in animals		
Yes	394	49.3
No	406	50.7
Knowledge of anthrax		
Adequate	464	58
Inadequate	336	42
Attitude towards anthrax		
Positive	424	53
Negative	376	47

### Livestock owner’s knowledge of anthrax

When participants were asked whether they knew the disease called anthrax, 98.3% of said “yes”. More than half (52%) knew anthrax could be transmitted from animals to humans, and about 42.7% knew the routes of transmission of the disease were consumption of the meat of dead animals (41.3%) and contacts with infected carcasses (6%). The majority (67.2%) of the farmers knew the treatment and control methods of the disease in animals and humans. For example, 91.4% of them pointed out that family members who got sick after consuming the carcass of anthrax killed animals should be taken to health facilities, while 7.7% recommended that the infected visit traditional healers. Over two thirds of the farmers knew the preventive measures of anthrax and indicated that burying and/or burning the carcass and vaccination of animals were among the most important measures (Table 2).

**Table 2:** Participant’s response to questions measuring knowledge of anthrax

Variables (Knowledge assessing questions)	Response	Frequency	Percent (%)
Do you know the disease called anthrax?	Yes	786	98.3
	No	14	1.7
Do you know the prevention methods of anthrax?	Yes	539	67.4
	No	261	32.6
Do you know anthrax can be transmitted from animal to human?	Yes	416	52
	No	384	48
Do you have knowledge about way of transmission?	Yes	342	42.7
	No	458	57.3
Do you know the treatment and control methods of the disease in animal and human?	Yes	538	67.2
	No	262	32.8
Do you have knowledge about carcass disposal method of anthrax?	Yes	181	22.6
	No	619	77.4

In general, about 58% (95%: 56.2–59.4%) of the respondents had adequate knowledge on anthrax.

### Attitude of respondents towards anthrax

Six questions were used to assess the attitude of participants towards anthrax, and 62.6 and 4.6% agreed and strongly agreed that anthrax was a health problem of the community, whereas about 19.5, 0.4 and 13% disagreed, strongly disagreed and I didn’t know that anthrax was a health problem of the community. Similarly, about 68.5% agreed and 11.5% strongly agreed that anthrax could be prevented by vaccination. The remaining, 1.5, 0.1 and 18.4% of the livestock owners disagreed, strongly disagreed and didn’t know that anthrax was prevented by vaccination.

Participants were asked whether burying or incinerating dead animals was one of the methods of controlling anthrax. The majority (33.4%) agreed, while 2.4% strongly agreed (Table 3).

**Table 3:** Participant’s response to questions measuring attitude towards anthrax

Questions used to assess attitude towards anthrax	Agree	Strongly agree	Disagree	Strongly disagree	Don’t know
Anthrax is a problem of the community	501(62.6%)	37(4.6%)	155 (19.5%)	3(0.4%)	104(13.0%)
Anthrax is prevented through vaccination	548(68.5%)	92(11.5%)	12(1.5%)	1(0.1%)	147(18.4%)
Anthrax can be controlled by treatment of sick animals	562(70.3%)	90(11.3%)	52(5.9%)	5(0.6%)	96(12.0%)
Anthrax is controlled by burying or incinerating of dead animals	267(33.4%)	19(2.4%)	216(27.0%)	1(0.1%)	297(37.1%)
Anthrax is transmitted through contacts with sick animals.	83(10.4%)	18(2.3%)	231(28.9%)	3(0.4%)	465(58.1%)
Consumption of meat that died from anthrax leads health risk.	358(44.8%)	52(6.5%)	102(12.8%)	5(0.6%)	288(36.0%)

Overall, 53% of the livestock owners had positive attitude towards anthrax.

### Anthrax prevention practices

We used seven questions to assess the anthrax prevention practices of the participants, of whom 52.8% said they took their animals to veterinary clinics, while 73% reported they used traditional medicine or healers. About three quarters (74%) of the livestock owners consumed the infected meat of animals that died from anthrax. Also more than three quarters (78%) used the skins and hides of the animals found dead with anthrax. Furthermore, 68.8% of the livestock owners used vaccinations to prevent anthrax (Table 4).

**Table 4:** Participant’s response to questions measuring anthrax prevention practices

Variable (Anthrax prevention practices assessing questions)	Response	Frequency	Percent (%)
Do you take sick animals to veterinary clinics?	Yes	422	52.8
	No	378	47.2
Do you burn or bury animals that died from anthrax?	Yes	7	0.9
	No	793	99.1
Do you use traditional medicine to treat sick animals?	Yes	584	73
	No	216	27
Do you use skin and hide of animals found dead with anthrax?	Yes	624	78
	No	176	22
Do you seek treatment at a health facility if anthrax is suspected?	Yes	441	55.1
	No	359	44.9
Do you vaccinate animals to prevent anthrax?	Yes	550	68.8
	No	250	31.2
Do you consume meat from animals that are suspected to have died from anthrax?	Yes	592	74
	No	208	26

In general, the right responses of the participants to the seven anthrax prevention practices assessing questions showed that only 25.4% (95%CI: 25.2–26.1%) were familiar with appropriate anthrax prevention practices.

### Factors associated with anthrax prevention practice

In the bivariable binary logistic regression analysis, twelve variables (attitude, knowledge, residence, health education on anthrax, availability of animal health care services, regulatory mechanisms, age, sex, educational level, occupation, previous infection experience of animals and humans) were included and tested (Additional file 1: Table S1). Of these, only four (attitude, health education, residence and educational status) were significantly associated with anthrax prevention practice in the multivariable analysis.

The result of the multivariable logistic regression analysis revealed that attitude towards anthrax, health education on anthrax, residence and educational status were significantly and independently associated with anthrax prevention practice among livestock owners. The odds of appropriate anthrax prevention practice were higher among livestock owners who had positive attitude compared to those who had negative attitude towards anthrax [adjusted odds ratio (AOR) = 4.16, 95% CI: 2.72, 6.37]. Likewise, livestock owners who received health education on anthrax were 2 times more likely to use appropriate anthrax prevention practices than those who didn’t get education [AOR = 2.00, 95% CI: 1.21, 3.28]. Livestock owners who had urban residence were 2.6 times more likely to practice appropriate anthrax prevention than those who lived in rural areas [AOR = 2.62, 95% CI: 1.43, 4.77]. The odds of appropriate anthrax prevention practice were higher among livestock owners who were able to read and write but had no formal education [AOR = 2.76, 95% CI: 1.74, 4.37] or completed primary [AOR = 3.6, 95% CI: 1.74, 4.37] or secondary school and above [AOR = 4.24, 95% CI: 1.61, 11.13] compared to those who were unable to read and write (Table 5).

**Table 5:** Factors associated with anthrax prevention practice in Sekota Zuria district, northeast Ethiopia, 2018

Variables	Anthrax prevention practice		Crude Odds Ratio (COR, 95% CI)	Adjusted Odds Ratio (AOR, 95% CI)
	Appropriate	Inappropriate		
Sex				
Female	47	125	1	
Male	155	473	1.15 (0.78–1.68)	
Age in years				
18–30	42	89	1	
31–41	75	233	0.65 (0.42–1.01)	
> = 42	85	276	0.96 (0.67–1.37)	
Residence				
Urban	59	40	5.7 (3.67, 8.87)	2.62 (1.43, 4.77)
Rural	144	557	1	1
Occupation				
Farmer	171	546	1.80 (0.78–4.14)	
Merchant	13	30	1.30 (0.46–3.69)	
Government employee	9	6	0.38 (0.10–1.40)	
Student	9	16	1	
Educational status				
Unable to read and write	53	375	1	1
No formal education, but able to read and write	69	148	3.3 (2.2, 4.95)	2.76 (1.74, 4.37)
Completed primary	50	59	6.00 (3.73, 9.63)	3.6 (2.01, 6.45)
Completed secondary or above	31	15	14.62 (7.41, 28.87)	4.24 (1.61,11.13)
Anthrax infection history in animals				
No	122	284	1	
Yes	80	314	1.69 (1.22–2.33)	
Attitude towards anthrax				
Positive	158	266	4.37 (3.02, 6.32)	4.16 (2.72, 6.37)
Negative	45	331	1	1
Health education about anthrax prevention				
Yes	64	61	4.05 (2.72, 6.02)	2.00 (1.21, 3.28)
No	139	536	1	1

## Discussion

The aim of this study was to assess the extent of anthrax prevention practices and associated factors among livestock owners in Sekota Zuria district. The study showed that a small proportion of farmers had appropriate anthrax prevention practices. Residence, educational status, attitude towards anthrax and health education about anthrax were the variables significantly associated with appropriate anthrax prevention practice.

Only 25.4% of the farmers utilized appropriate anthrax prevention practices in the district. Studies conducted to assess anthrax prevention practices among livestock owners in Turkey and Zimbabwe indicated that 51.9% [13] and 86% [3], of the owners appropriately managed the disease, respectively. Compared to these findings, the result of this work was lower. The observed discrepancy might be due to socio-economic differences among the settings. In Ethiopia, Sekota Zuria district is one of the most drought affected area, where household food insecurity is high compared to the two countries. This may drive the local population to consume the meat of dead animals. In addition, little attention has been given to animal health care services by the concerned bodies despite the fact that Ethiopia is the leading country in livestock assets. The huge numbers of animals Ethiopians possess challenge the capacity of owners to implement appropriate interventions as opposed to farmers in Turkey and Zimbabwe.

Positive attitude towards the prevention of the disease was one of the main factors identified to have a significant association with the anthrax prevention practice. This might be related to the fact that human behaviour is influenced by perceptions and attitude which are the driving forces for activities [13]. This study implied that creating positive attitude towards anthrax transmission, prevention and its public health importance among livestock owners is vital in the prevention of the disease. The current finding was in agreement with those of other studies reported from Turkey, Kenya and Tanzania, and found out that livestock owners with low level of awareness were less likely to use protective clothing when dealing with abortions or calves with diarrhoea and during on-farm activities. Thus, they were exposed to an increased risk for contracting zoonosis [13–15].

The odds of appropriate anthrax prevention practice were higher among livestock owners who received health education about anthrax prevention practices compared to those who didn’t receive. That is, getting health education about anthrax prevention practices from animal health workers is one of the proven interventions of boosting the knowledge and perceptions of livestock owners or raising their awareness of zoonotic diseases and help them use appropriate anthrax prevention practices. This finding suggests that providing continuous health education for livestock owners has a vital role in improving anthrax prevention practices in their areas. In line with this, a previous study indicated that lack of appropriate health education and economic problems may tend to encourage people to consume raw or undercooked animal products infected with anthrax [16].

The multivariate analysis revealed that livestock owners who lived in urban areas were 2.6 times more likely to employ appropriate anthrax prevention practices compared to rural dwellers. This might because urban dwellers have better access to information and veterinary services which enable them to prevent anthrax appropriately. In addition, urban dwellers are more likely to get animal health care services since most vaccinations and information campaigns focus on areas with better infrastructure, such as roads [17]. This observation is supported by previous reports which reveal that the risk for zoonosis is significantly higher among rural farms compared to peri or urban ones (*P* < 0.05), and rural communities commonly consume raw milk, meat and blood [15, 18, 19] .

Respondents who were able to read and write but had no formal education or had formal education were more likely to prevent anthrax better than livestock owners who were unable to read and write. Education noticeably helps to enhance awareness thereby enabling owners to develop good behaviours and prevent the disease. This indicates that educating people is of paramount importance in increasing the proportion of livestock owners with appropriate anthrax disease prevention practices. Other earlier studies also reported similar findings [17, 20, 21].

This study revealed the extent of anthrax prevention practices and its associated factors among the most vulnerable population groups, farmers in rural areas of Ethiopia, where there is a scarcity of literature. Hence, this study can serve as a baseline for future studies since there is limited evidence on anthrax prevention practices in the country.

The cross-sectional design of the study might have limited its capacity to measure the cause-effect relationships of the outcome and potential correlates. In addition, the study might be prone to recall bias since some of the information collected was based on events that happened sometime in the past. Finally, information on some variables, such as wealth index, the presence of collaborative and preventive activities of both human and animal health workers, housing of livestock and participants’ quarantine practices of domestic animals suspected to have or have been exposed to anthrax were not addressed in this study.

## Conclusion

In Sekota Zuria district, only one-quarter of the livestock owners appropriately prevented anthrax. Level of education, residence, health education on anthrax prevention and attitude towards the disease were found to be significantly associated with the prevention practice. Thus, providing continuous health education and creating awareness on the prevention by veterinary professionals would be crucial to improve anthrax prevention practices of livestock owners. Furthermore, providing effective formal education to farmers is essential to curb the problem in the region.

## Methods

### Study design and setting

A community-based cross-sectional study was conducted from March 25 to April 20, 2018, in Sekota Zuria one of the seven districts of Wag-Himra Zone, located in the Amhara Regional State, Ethiopia. The district is located 460 km northeast of Addis Ababa, the capital of Ethiopia. Its altitude varies from 1340 to 2200 m above the mean sea level, with an annual rainfall ranging from 350 to 700 mm, mainly from July to September. The pattern and distribution of the rainfall is erratic and uneven. The average temperature ranges from 16 to 27 °C. The district consists of 35 kebeles *(The smallest administrative units in Ethiopia)* with an estimated total population of 173,026. According to the district administration, the total estimated livestock population was 405,373 (100,515 bovines, 179,914 ovine and caprine, 20,053 equines and 104,891 poultry) served by 21 veterinary clinics.

### Study population, sample size determination, sampling techniques and procedures

All livestock owners aged 18 years and above and lived in the district for at least six months were included in the study. Since there has been no previous study on the anthrax prevention practices, the sample size was calculated by considering the assumptions of 50% prevalence, 95% confidence interval, 5% margin of error, 10% non-response rate and a design effect of 2 using Epi-Info version 7 which yielded 844 livestock owners. Seven of the 35 kebeles in Sekota Zuria district, namely kebele 01, 03, 08, 13, 20, 24 and 31 were selected using the lottery method. According to evidence from the district office and the initial assessment done, there were 8033 households with livestock in the seven kebeles. The baseline assessment indicated that there were 1250, 1300, 1075, 1138, 1225, 1085, and 960 livestock owning households in kebele 01, 03, 08,13, 20, 24, and 31, respectively.

Proportional allocation was used to select representative samples (households) from each kebele. The systematic random sampling method was used to select 844 livestock owners from 844 households. In addition, sampling interval (K) was determined and found to be 10. Then, a list of numbers from 1 to 10 was prepared and 3 was drawn as the first household/farmer to be interviewed. Accordingly, households were selected at every 10th interval, and farmers in the selected households were interviewed. The number of participants included from 01, 03, 08,13, 20, 24, and 31 kebeles by proportion were 131, 137, 113, 119, 129, 114, and 101, respectively.

### Operational definitions and study variables

#### Anthrax prevention practice

The study participants were deemed to have respective appropriate or inappropriate anthrax prevention practices if they were able to answer more than or equal to 50% and less than 50% correct responses out of the seven questions [13]. It was measured using the following seven questions. Do you take sick animals to veterinary clinics? Do you burn or bury animals that died from anthrax? Do you use traditional medicine to treat sick animals? Do you use the skins and hides of animals found dead with anthrax? Do you seek treatment at a health facility if anthrax is suspected? Do you vaccinate animals to prevent anthrax? And do you consume the meat of animals that are suspected to have died from anthrax?

#### Attitude towards anthrax

It was measured through six attitude assessing questions, using the Likert scale. These questions were; anthrax is a problem of the community; it is prevented through vaccination; it is controlled through treatment of sick animals; it is controlled through burial or incineration of dead animals; it is transmitted through contacts with sick animals and the consumption of meat of animals that died from anthrax leads to health risk. Accordingly, participants’ responses were aggregated in to agree and disagree and were considered as having positive attitude if they were able to correctly respond more than or equal to 50% on the six attitude assessing questions; otherwise, they were considered as having negative attitude [13].

#### Knowledge of anthrax

It was also measured using the following six questions. Do you know the disease called anthrax? Do you know the prevention methods of anthrax? Do you know that anthrax can be transmitted from animals to humans? Do you have knowledge about the ways of transmission? Do you know the treatment and control methods of the disease in animals and humans? And do you have knowledge about carcass disposal methods? Thus, the knowledge of respondents was classified as adequate and inadequate if they were able to correctly answered more than or equal to 50% and less than 50% from the six knowledge assessing questions, respectively [13].

Anthrax prevention practice was the dependent variable. Data on independent variables such as, socio-demographic characteristics (age, sex, occupation, educational level, residence and knowledge of anthrax, experience of infection in animal/human, getting health education on anthrax and regulatory mechanism/veterinary supervision/screening system were collected.

### Data collection procedures and quality control

The questionnaire was originally prepared in English and translated to Amharic and back to English to ensure consistency. The data collectors and supervisors were trained on quality, informed consent and interviews. The questionnaire was pretested on 5% of the total sample on similar participants and setting outside the actual study area, and modifications were made on the arrangement, and some questions were rewritten to make them clearer. The principal investigator who daily monitored the data collection process and the supervisors spot-checked and reviewed the completed copies of the questionnaires for matters of completeness and consistency.

### Data processing and analysis

Data was checked for completeness, cleaned and then entered into Epi-Info version 7 and exported to Statistical Package for Social Sciences (SPSS) version 22 for analysis. Descriptive statistics, including frequency tables, proportions and texts were used to summarize and present the findings. The knowledge score was computed using six assessment questions, and those who scored 50% and more were considered as “having adequate knowledge”; otherwise, they were taken as having “inadequate knowledge”. Attitude score was also calculated by computing the responses given to the six attitude measuring questions. Accordingly, respondents who scored 50% and above were classified as having “positive attitude” and those who scored below 50% were considered as having “negative attitude” towards anthrax. Seven questions were used to measure anthrax prevention practice. Responses were computed and participants who scored 50% and above were categorized as having “appropriate anthrax prevention practice”, and below 50% scorers were categorized as having “inappropriate anthrax prevention practice”. A value of “1” was assigned to adequate knowledge, positive attitude, and appropriate anthrax prevention practice and “0” (zero) to inadequate knowledge, negative attitude and inappropriate anthrax prevention practice. The bivariate analysis was used to check the associations between independent variables and anthrax prevention practice. In the first place, the association of each variable with the dependent variable was tested. Independent variables included in the bivariate analysis were sex, age, educational status, residence, occupation, knowledge of anthrax, attitude towards anthrax, health education about anthrax, presence of animal health care services, anthrax infection history in animals, presence of anthrax regulatory mechanisms/surveillance systems, and anthrax infection history in humans. In the second step, the multivariable logistic regression was computed to test the presence of associations. All independent variables with 0.2 and less *p*-values during the bivariate analysis were included in the multivariable logistic regression model to control possible effects of confounders. Sex, age, educational status, residence, occupation, attitude towards anthrax, health education about anthrax and anthrax infection history in animals were the independent variables included in the final model. Finally, variables with *p*-values of less than 0.05 at a 95% CI were considered statistically significant in the multivariable analysis.

## Supplementary information

**Additional file 1: Table S1.** Bivariate analysis of variables associated with anthrax prevention practice in among livestock owners in Sekota Zuria district, northeast Ethiopia. This data/table shows the independent variables associated with anthrax prevention practice, included during bivariable analysis among livestock owners in Sekota Zuria district.

## Data Availability

The datasets used and/or analysed during the current study are available from the corresponding author on reasonable request.

## Abbreviations

AOR: Adjusted Odds Ratio
CI: Confidence Interval
COR: Crude Odds Ratio
SPSS: Statistical Package for Social Science
