# Supplementary material for: Livestock owners’ anthrax prevention practices and its associated factors in Sekota Zuria district, Northeast Ethiopia
Source: BMC Vet Res. 2020 Feb 3;16:39. doi: 10.1186/s12917-020-2267-0 (PMC6998812; doi:10.1186/s12917-020-2267-0)
Supplement: Supplementary file 1 — Additional file 1: Table S1. Bivariate analysis of variables associated with anthrax prevention practice in among livestock owners in Sekota Zuria district, northeast Ethiopia. This data/table shows the independent variables associated with anthrax prevention practice, included during bivariable analysis among livestock owners in Sekota Zuria district. [file 12917_2020_2267_MOESM1_ESM.docx]

**Table 1: Bivariate analysis of variables associated with anthrax prevention practice in among livestock owners in Sekota Zuria district, northeast Ethiopia**

| **Variables** | **Anthrax prevention practice** | | **Crude odds ratio (COR, 95%CI)** | **P-value** |
| --- | --- | --- | --- | --- |
|  | **Good** | **Poor** |  |  |
| **Sex** | | | | |
| Female | 47 | 125 | 1 |  |
| Male | 155 | 473 | 1.15 (0.78-1.68) | 0.18 |
| **Age** | | | | |
| 18-30 | 42 | 89 | 1 |  |
| 31-41 | 75 | 233 | 0.65 (0.42-1.01) | 0.06 |
| >=42 | 85 | 276 | 0.96 (0.67-1.37) | 0.81 |
| **Occupation** | | | | |
| Farmer | 171 | 546 | 1.80 (0.78-4.14) | 0.17 |
| Merchant | 13 | 30 | 1.30 (0.46-3.69) | 0.62 |
| Government employed | 9 | 6 | 0.38 (0.10-1.40) | 0.14 |
| Student | 9 | 16 | 1 |  |
| **Residence** | | | | |
| Urban | 59 | 40 | 5.7 (3.67-8.87) | 0.001 |
| Rural | 144 | 557 | 1 |  |
| **Anthrax infection history in animals** | | | | |
| Yes | 80 | 314 | 1.69 (1.22-2.33) | 0.002 |
| No | 122 | 284 | 1 |  |
| **Presence of animal health care service** | | | | |
| Yes | 200 | 592 | 0.98 (0.19-4.93) | 0.99 |
| No | 2 | 6 | 1 |  |
| **Presence of regulatory mechanisms/anthrax surveillance** | | | | |
| Yes | 55 | 183 | 1.18 (0.83-1.68) | 0.37 |
| No | 147 | 415 | 1 |  |
| **Anthrax infection history in man** | | | | |
| Yes | 43 | 125 | 0.97 (0.66-1.44) | 0.91 |
| No | 159 | 473 | 1 |  |
| **Knowledge of anthrax** | | | | |
| Good | 113 | 351 | 1.12 (0.81-1.54) | 0.49 |
| Poor | 89 | 247 | 1 |  |
| **Attitude towards anthrax** | | | | |
| Good | 158 | 266 | 4.37 (3.02-6.32) | 0.001 |
| Poor | 45 | 331 |  |  |
| **Educational level** | | | | |
| No read and write | 53 | 375 | 1 |  |
| Read and write | 69 | 148 | 3.3 (2.2, 4.95) | 0.038 |
| Primary education | 50 | 59 | 6.00 (3.73, 9.63) | 0.003 |
| Secondary and above | 31 | 15 | 14.62 (7.41, 28.87) | 0.0001 |
| **Health education about anthrax prevention** | | | | |
| Yes | 64 | 61 | 4.05 (2.72, 6.02) | 0.001 |
| No | 139 | 536 | 1 |  |
